# Comprehensive pan-cancer analysis on CBX3 as a prognostic and immunological biomarker

**DOI:** 10.1186/s12920-022-01179-y

**Published:** 2022-02-16

**Authors:** Hongjuan Niu, Peiqiong Chen, Lu Fan, Boyu Sun

**Affiliations:** 1 School of Pharmacy in Minzu University of China, Beijing, 100081 China; 2 Department of Pharmacy in Zhengzhou Ninth People’s Hospital, Zhengzhou, 450000 China; 3 The Third People’s Hospital of Qingdao, Qingdao, 266000 China

**Keywords:** CBX3, Pan-cancer, Expression, Prognosis, Immune

## Abstract

**Background:**

Increased evidence supports the relationship between chromobox protein homolog 3 (CBX3) and tumorigenesis of some cancers. However, the role of CBX3 in pan-cancers remains poorly defined. In the research, we aimed to investigate the prognostic value and the immunological functions of CBX3.

**Results:**

We explored the potential oncogenic roles of CBX3 in mRNA and protein levels based on the diverse databases, including the expression, the correlation with prognosis, tumor microenvironment (TME), DNA methylation, protein phosphorylation and enrichment analysis across all TCGA tumors. The results show that CBX3 is overexpressed in multiple cancers, and significant correlations exist between high expression and adverse prognosis in most tumor patients. We observed an enhanced phosphorylation level in uterine corpus endometrial carcinoma, colon cancer and lung adenocarcinoma. A distinct relationship was also found between CBX3 expression and TME, including immune infiltration of tumor-infiltrating lymphocytes and cancer-associated fibroblasts, immune score or matrix score, immune checkpoints. The correlative transcription factors and miRNAs of CBX3-binding hub genes were analyzed to investigate the molecular mechanism. Moreover, alcoholism and alteration of DNA cellular biology may be involved in the functional mechanisms of CBX3.

**Conclusion:**

The first pan-cancer study offers a relatively comprehensive cognition on the oncogenic roles of CBX3 as a prognostic and immunological marker in various malignant tumors.

**Supplementary Information:**

The online version contains supplementary material available at 10.1186/s12920-022-01179-y.

## Introduction

Tumorigenesis is a complex process involving the immune system, where TME plays a crucial role. Due to the elusive regulation mechanism, the interaction between tumor and immune remains a focus. It is urgent to conduct a pan-cancer analysis of genes to assess the value for clinical prognosis and potential therapeutic targets. The expression pattern, survival analysis, and tumor immune infiltration of genes in different tumors across numerous databases, including the available TCGA project, GEO database, cBioPortal, GEPIA, and TIMER database [1–3], can allow us to conduct pan-cancer analysis.

CBX3 is a member of the heterochromatin protein 1 (HP1) family. The protein encoded by this gene binds DNA and composes heterochromatin. CBX3 has several synonyms, including HECH, HP1γ, heterochromatin protein 1 homolog gamma, heterochromatin-like protein 1and modifier 2 protein. Moreover, together with CBX1 and CBX5, the three belong to HP1 orthologs. It is reported that CBX3 plays a principal role in transcriptional repression or activation, epigenetic modification, as well as cell differentiation [4, 5]. Additionally, it was abnormally expressed and dysregulated in many cancers. Currently, the expression of CBX3 was found to increase in non-small cell lung cancer [6], prostate cancer [7], colorectal cancer [8], and breast cancer [9], which may predict the poor prognosis of patients. Some literature reported that CBX3 was related to immunity in specific cancers. On this account, CBX3 may potentially be of great importance in the epigenetic regulation of cancer development. In addition, it is expected to be a therapeutic target for cancers. Nowadays, CBX3 has obtained widespread attention as a potential biomarker in several cancers [10]. However, the expression, function, and mechanism of CBX3 in various tumors remain unknown.

In this study, we conducted a pan-cancer analysis of CBX3 based on multiple databases. Some factors, such as gene expression and alteration, clinical prognosis, protein phosphorylation, TME, and relevant cellular pathway, were involved. Based on the results, we aimed to investigate the value as a biomarker and to evaluate the potential molecular mechanism of CBX3 in different cancers.

## Methods

### Gene expression analysis

In the “Gene_DE” module of TIMER2.0 (tumor immune estimation resource, version 2) (http://timer.cistrome.org/) [11], CBX3 was searched and observed the expression discrepancy between tumor and adjacent normal tissues for the various tumors in the TCGA project. The ONCOMINE database (https://www.ONCOMINE.org/) [12] integrates RNA and DNA-sequence data from GEO, TCGA, and published literature. We entered CBX3 in the search box, then set the P value (0.01), fold change (1.5), gene rank (10%), and data type (mRNA) to get the differential expression data of CBX3 in various tumors. The red indicates overexpression, and the blue is the opposite. Besides, the dark color and the large value were representatives of the high expression amount. GEPIA2 (Gene Expression Profiling Interactive Analysis, version 2) webserver (http://gepia2.cancer-pku.cn/#analysis) [13] combines TCGA and GTEx database. To compare the expression of CBX3 in cancer tissues and corresponding normal tissues, we used the “Expression analysis -Box Plots” module of the GEPIA2 to obtain box plots of the expression difference of the GTEx (Genotype-Tissue Expression) database. The settings included *P* value cutoff = 0.01, log2FC (fold change) cutoff = 1, and “Match TCGA normal and GTEx data”. In addition, on the base of the “Pathological Stage Plot” module of HEPIA2, the violin plots of the CBX3 expression in different pathological stages (stage I, stage II, stage III, and stage IV) of all TCGA tumors were obtained. The log2 [TPM (Transcripts per million) + 1] transformed expression data were applied for the box or violin plots.

The UALCAN portal (http://ualcan.path.uab.edu/analysis-prot.html) can be used to conduct protein expression analysis of the CPTAC (Clinical proteomic tumor analysis consortium) dataset [14]. With the help of the website, we explored the expression of the total protein or phosphoprotein of CBX3 between primary tumor and normal tissues, respectively. The Human Protein Atlas (https://www.proteinatlas.org/) is a database about gene immunohistochemistry in cancer. The distribution and expression of each protein in 48 kinds of normal human tissues and 20 types of tumor tissues were examined by immunohistochemical technique, attached with at least 576 staining pictures.

### Survival prognosis analysis

In the part, considering that there may be other factors result in death except tumor during the follow-up, we also visualized the relationship between CBX3 expression and prognosis (OS: overall survival; DSS: disease-specific survival; DFI: disease-free interval; PFI: progression-free interval) in the form of Kaplan–Meier curves via the Sangerbox online platform (http://www.sangerbox.com/tool). The univariate survival analysis was applied to calculate the hazard ratio (HR) and 95% confidence intervals.

### DNA methylation analysis

DNMIVD (http://119.3.41.228/dnmivd/index/) [15] is a database of methylation pan-cancer analysis based on the methylation chip database of TCGA and GEO. This database can view the methylation results of a gene in pan-cancer, and understand the influence of certain methylation sites on prognosis models. After search of CBX3, the basic information of methylation and probe related to the gene was obtained. The methylation difference of this gene in the promoter region of target cancer was shown in a box diagram. Spearman correlation was used to analyze the relationship between methylation and expression. The relationship between methylation and prognosis, including OS, DFI and PFI was grouped according to the median value in Kaplan–Meier plots.

### Genetic alteration analysis

In the cBioPortal web (https://www.cbioportal.org/) [16], we entered “CBX3” in the “TCGA Pan-Cancer Atlas Studies” section for queries of the genetic alteration characteristics across all TCGA tumors. In the “Mutations” module, the mutated site information was displayed in the schematic diagram of the 3D (three-dimensional) structure. Moreover, we also applied the “Comparison” module to obtain the data on the overall, disease-free, progression-free, and disease-free survival differences for the cancer cases. The corresponding Kaplan–Meier plots with the log-rank *P* value were generated for the significantly different cancers with or without CBX3 genetic alteration.

### Protein phosphorylation analysis

To obtain a schematic diagram of the CBX3 protein domain, first of all, we used the UniProt tool (https://www.uniprot.org/) [17] to find ID of the corresponding protein. Subsequently, with the help of the SMART (http://smart.embl-heidelberg.de/) tool, we acquired the protein domains. The UALCAN portal was applied to display the phosphorylation site and protein expression level of CBX3 across TCGA cancers.

### TME analysis

The TME analysis included the evaluation on the immune infiltration of tumor-infiltrating lymphocytes (TILs) and cancer-associated fibroblasts (CAFs), the immune score or matrix score, and expression of immune checkpoint markers. With the help of Sangerbox-pan-cancer analysis tool, the correlation between CBX3 expression and the immune infiltration of the TILs (B cells, CD4 + T cells, CD8 + T cells, neutrophils, macrophages and dendritic cells) across TCGA cancers were evaluated. Furthermore, the top 3 tumors with significant differences were displayed. In addition, we analyzed the relationship between CBX3 expression and the immune score or matrix score in the form of StromalScore, Est_ImmuneScore, and ESTIMATEScore. Similarly, the scatter plots presented the top 3 tumors with significant differences, respectively. Subsequently, we estimated the correlation between the expression levels of 40 common immune checkpoint markers and CBX3 expression by Spearman and Pearson correlation analyses. The levels of gene expression were shown as log2 RSEM values.

With the help of the TIMER2.0 web server, we explored the association between CBX3 expression and immune infiltrates in the “Immune-Gene” module. The CAFs were selected in the immune infiltrates part. We set the TIMER, EPIC, MCP-COUNTER, and TIDE algorithms for estimations. Similarly, we applied the “Immune-Outcome” module to explore the clinical relevance of tumor immune subsets. We selected the immune cells of the CAFs. The age and stage were searched in the clinical part, respectively. Ultimately, we got the correlation heatmap and scatter plot of CBX3 and CAFs in gene expression and clinical outcome.

### Correlation analysis of CBX3-binding hub genes

We first searched the STRING website (https://string-db.org/) according to the keywords “CBX3” and “Homo sapiens”. Afterward, the main parameters were set as follows: Low confidence (0.150), evidence, no more than 50 interactors, and experiments. Subsequently, we screened 50 CBX3-binding genes that were experimentally verified. Ultimately, the data was exported to Cytoscape v3.8.0 software for modification of the picture. Simultaneously, we obtained 10 hub ones from CBX3-binding genes via the Cytohubba module.

In the NetworkAnalyst website (https://www.networkanalyst.ca/), the protein-chemical interaction was identified by the Comparative Toxicogenomics Database (CTD) (downloaded on Nov. 2016) [18] to identify potential chemical material that could influence CBX3. In a sense, it can work in the prevention of diseases caused by CBX3 in advance. To identify transcription factors (TFs) that regulate the hubs at a transcriptional level, TF-hub interactions were obtained via the JASPAR database [19]. The topological parameters (degree and betweenness centrality) were identified via NetworkAnalyst [20]. Similarly, to identify regulatory miRNAs that influence hubs at the post-transcriptional level, miRNAs-gene interactions were gained from TarBase [21]. The database included experimentally supported miRNA-gene interrelation, and topological parameters were analyzed using NetworkAnalyst [20].

### CBX3-related genes enrichment analysis

In the “Similar Gene Detection” module of GEPIA2, we obtained the top 100 CBX3-correlated target genes. In addition, the “correlation analysis” module was applied to perform Pearson correlation analysis for five pairwise genes of CBX3, respectively. Then, we acquired five corresponding dot plots marked with the *P* value and the correlation coefficient (R). Moreover, based on the “Gene_Corr” module in the “Exploration” of TIMER2.0, we obtained the heatmap data of the five genes in TCGA cancers.

The venny2.1.0 web servicer (https://bioinfogp.cnb.csic.es/tools/venny/index.html) was applied to conduct an intersection analysis on the CBX3-binding and interacted genes. Moreover, we combined the two sets of data to perform Gene Ontology (GO) enrichment analysis and Kyoto encyclopedia of genes and genomes (KEGG) [22] pathway analysis with R package (clusterProfiler, enrichplot, and ggplot2). The data for biological process (BP), cellular component (CC), and molecular function (MF) in the GO enrichment analysis were visualized as bubble chart. The R language software [R-4.0.2, 64-bit] (https://www.r-project.org/) was used in the process. Two-tailed *P* < 0.05 was considered statistically significant.

## Results 

### The expression level of CBX3 in pan-cancer

The relationship between human CBX3 (NM_007276.4 for mRNA or NP_009207.2 for protein) and pan-cancer was explored from many aspects in the study. We analyzed the distribution and expression of CBX3 in different tumor cells and tissues based on the combination of the TCGA, GTEx, and HPA datasets.

**Fig. 1:**
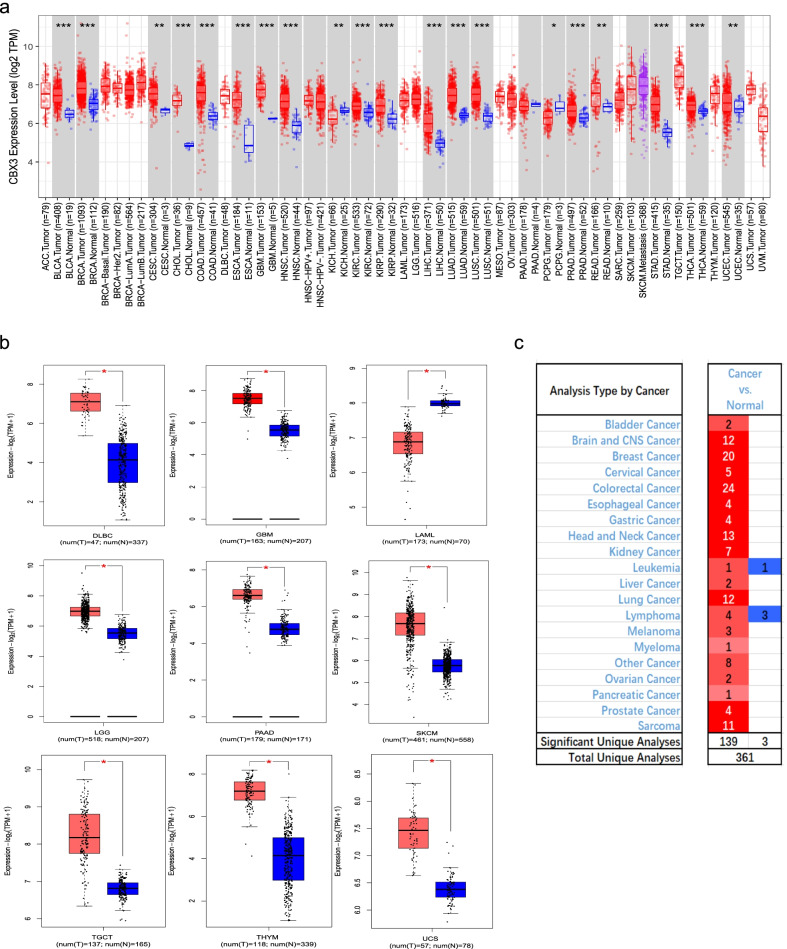
The expression level of CBX3 gene in different tumors in multiple databases. **a** The gene expression of CBX3 in different cancers in TCGA database was analyzed through TIMER2.0. *P < 0.05; **P < 0.01; ***P < 0.001. **b** For the absent types of cancers in TCGA, the corresponding normal tissues of the GTEx database were included as controls. The box plots were supplied. *P < 0.05. **c** Transcriptional expression of CBX3 in different types of cancer diseases (ONCOMINE database). The difference in transcriptional expression was compared by students’ t-test

We applied the TIMER2.0 tool to analyze the expression of CBX3 in cancers of TCGA. As shown in Fig. 1a, CBX3 in most tumor tissues has a higher expression level, compared with that in normal tissue. The tumors include BLCA (Bladder urothelial carcinoma), BRCA (Breast invasive carcinoma), CHOL (Cholangiocarcinoma), COAD (Colon adenocarcinoma), ESCA (Esophageal carcinoma), GBM (Glioblastoma multiforme), HNSC (Head and neck squamous cell carcinoma), KIRC (Kidney renal clear cell carcinoma), KIRP (Kidney renal papillary cell carcinoma), LIHC (Liver hepatocellular carcinoma), LUAD (Lung Adenocarcinoma), LUSC (Lung squamous cell carcinoma), PRAD (Prostate adenocarcinoma), STAD (Stomach adenocarcinoma), THCA (Thyroid carcinoma) (*P* < 0.001), CESC (Cervical squamous cell carcinoma and endocervical adenocarcinoma), UCEC (Uterine corpus endometrial carcinoma) (*P* < 0.01). While the KICH (Kidney chromophobe) (*P* < 0.01), and PCPG (Pheochromocytoma and paraganglioma) (*P* < 0.05) have lower expression level of CBX3 than the corresponding control tissues.

With the help of GEPIA 2 webserver, we combined TCGA and GTEx databases to compare gene expression in tumor tissues and normal ones. The expression difference of CBX3 in some tumors were further evaluated (Fig. 1b), involving SKCM (Skin cutaneous melanoma), GBM, LAML (Acute myeloid leukemia), DLBC (Lymphoid neoplasm diffuse large B-cell lymphoma), LGG (Brain lower grade glioma), PAAD (Pancreatic adenocarcinoma), TGCT (Testicular germ cell tumors), UCS (Uterine carcinosarcoma) and THYM (Thymoma) (*P* < 0.05). However, for ACC (Adrenocortical carcinoma), SARC (Sarcoma), OV (Ovarian serous cystadenocarcinoma), and CESC (Additional file 1: Fig. S1), it showed no significant difference.

To analyze the mRNA expression of CBX3 in cancer patients, the ONCOMINE database was used. As were shown in Fig. 1c, mRNA expressions of CBX3 in various types of cancers were measured and compared to normal tissues. We found significantly increased mRNA expression of CBX3 in multiple tumor tissues.

**Fig. 2:**
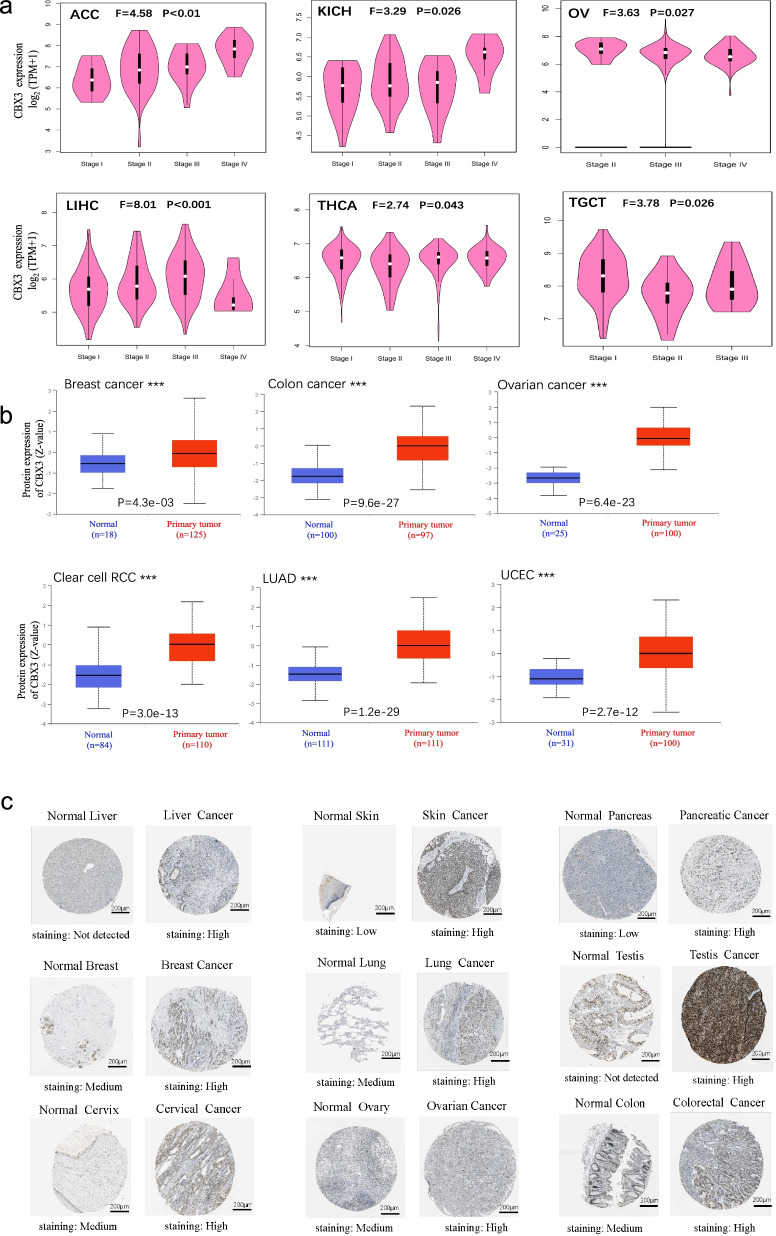
**a** Based on the TCGA dataset, the expression levels of CBX3 were analyzed by the main pathological stages (stage I, stage II, stage III, and stage IV) of ACC, KICH, OV, LIHC, THCA, and TGCT. Log2 (TPM + 1) was applied for log-scale. **b** Based on the CPTAC dataset, we analyzed the expression level of CBX3 total protein between normal tissue and primary tissue of breast cancer, colon cancer, ovarian cancer, clear cell RCC, LUAD, and UCEC. ***P < 0.001. **c** We showed representative and contrastive immunohistochemistry images of CBX3 in tumor tissues and normal tissues

In order to clarify the expression level of CBX3 in pathological stages of different tumors, we applied the “Pathological Stage Plot” module of GEPIA2 tool. Among them, the cancers including LIHC (*P* < 0.001), ACC (*P* < 0.01), THCA, TGCT, KICH, OV (*P* < 0.05) showed a difference between gene expression and pathological stages (Fig. 2a).

The results of the CPTAC dataset showed compared with the normal tissues, the primary tumors of breast cancer, colon cancer, ovarian cancer, clear cell RCC, LUAD, and UCEC had higher expression of CBX3 total protein (Fig. 2b, *P* < 0.001). Moreover, the additional analysis results in the ONCOMINE database (Fig. 1c) further confirmed that CBX3 is highly expressed in breast cancer, cervical cancer, lung cancer, kidney cancer, colorectal cancer, and ovarian (*P* < 0.01), compared with normal controls.

We tried to explore the protein expression patterns of CBX3 in cancers by the Human Protein Atlas. As shown in Fig. 2c, CBX3 proteins were not detected in normal liver and testis tissues, whereas low expressions were observed in normal skin and pancreas tissues. In addition, medium protein expressions were showed in normal tissues of breast, lung, cervix, ovary, and colon. While high expressions in their corresponding cancers were observed. Overall, the results showed that genic and proteinic terms of CBX3 were overexpressed in most cancers.

Data from multiple databases showed that mRNA and protein expression of CBX3 in tumor tissues was significantly increased. It indicated thatCBX3 might function as an oncogenic molecule in the development of diverse tumors.

### Prognostic value of CBX3 in pan-cancer

**Fig. 3:**
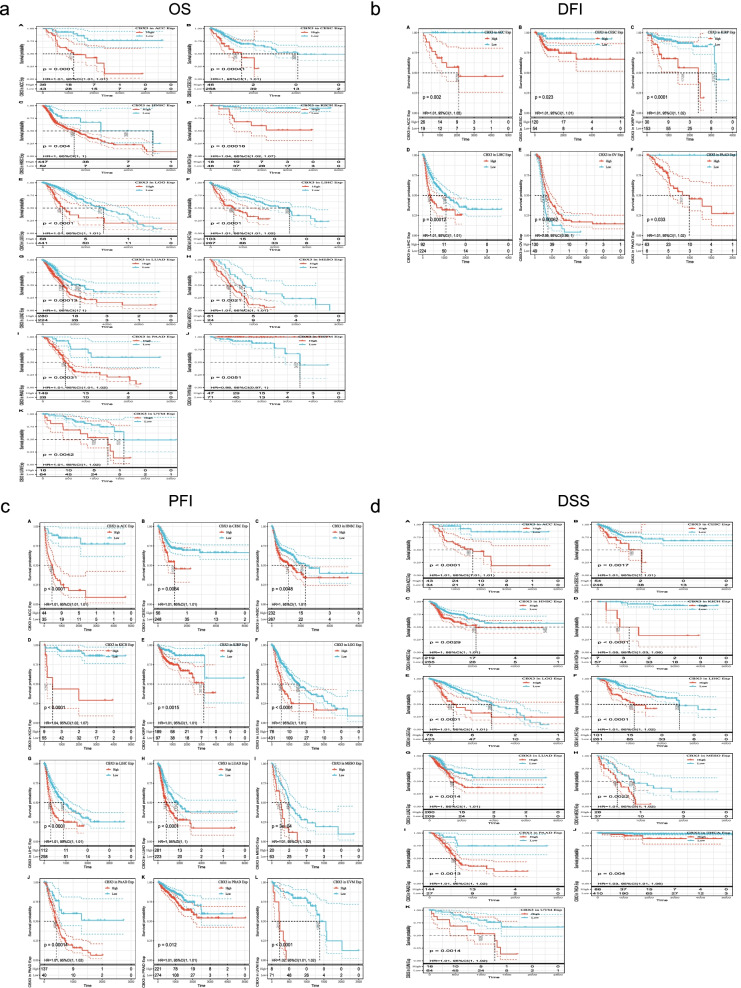
Correlation between CBX3 gene expression and survival prognosis of cancers in TCGA. The Kaplan–Meier curves of OS (**a**), DFI (**b**), PFI (**c**), and DSS (**d**) in different tumors with positive results are offered

We evaluated the effect of CBX3 expression on prognostic value for the patients in pan-cancer. According to the expression levels of CBX3, the cancer cases were divided into high-expression and low-expression groups. Notably, CBX3 expression was significantly correlated with OS in 11 types of cancer (ACC, CESC, HNSC, KICH, LGG, LIHC, LUAD, MESO, PAAD, THYM, and UVM) (Table 1, Fig. 3a). In addition, except as a protective factor in THYM, CBX3 appeared to be a risk factor in other cancer types. Subsequently, we investigated the relationship between CBX3 expression and DFI. The results revealed that increased gene expression was correlated with poor prognosis in ACC, CESC, KIRP, LIHC, and PAAD, while with favorable prognosis in OV (Table 2, Fig. 3b). Next, the relationship between expression and PFI in cancers from TCGA was analyzed. Results showed CBX3 expression impacted patients’ PFI in 12 cancer types (Table 3). Specifically, the Kaplan–Meier curves of all the cancers (Fig. 3c) showed that high expression of CBX3 was significantly correlated with poor prognosis of patients in ACC, CESC, HNSC, KICH, KIRP, LGG, LIHC, LUAD, MESO, PAAD, PRAD, and UVM. Ultimately, we assessed the relationship between CBX3 expression and DSS to exclude non-tumor death factors. The results showed that high expression of CBX3 affected DSS unfavorably in ACC, CESC, HNSC, KICH, LGG, LIHC, LUAD, MESO, PAAD, THCA, UVM (Table 4, Fig. 3d). In conclusion, these results indicated that CBX3 expression level is a key influencing factor for the prognosis of patients, especially those with ACC, CESC, LIHC, and PAAD. Moreover, the above data from TCGA revealed that elevated expression of CBX3 is associated with the poor prognosis of cases in most tumors.

**Table 1:** The correlation between CBX3 and OS in cancers

Kind of cancer	*P* value	HR
THYM	1.7e-02	0.98
ACC	2.7 e-07	1.01
CESC	1.4e-02	1.00
HNSC	2.0e-02	1.00
KICH	1.7e-04	1.04
LGG	4.6e-07	1.01
LIHC	3.6e-07	1.01
LUAD	3.4e-03	1.00
MESO	2.8e-02	1.01
PAAD	2.8e-04	1.01
UVM	6.3e-03	1.01

**Table 2:** The correlation between CBX3 and DFI in cancers

Kind of cancer	*P* value	HR
ACC	0.0021	1.01
CESC	0.0400	1.01
KIRP	0.0140	1.01
LIHC	0.0130	1.01
PAAD	0.0280	1.01
OV	0.0031	0.99

**Table 3:** The correlation between CBX3 and PFI in cancers

Kind of cancer	*P* value	HR
ACC	9.9e-10	1.01
CESC	5.0e-03	1.01
HNSC	1.8e-03	1.00
KICH	3.1e-04	1.04
KIRP	1.1e-02	1.01
LGG	3.0e-07	1.00
LIHC	1.5e-04	1.01
LUAD	1.7e-03	1.00
MESO	1.8e-02	1.01
PAAD	4.2e-04	1.01
PRAD	3.5e-03	1.01
UVM	1.1e-04	1.02

**Table 4:** The correlation between CBX3 and DSS in cancers

Kind of cancer	*P* value	HR
ACC	3.1e-07	1.01
CESC	3.9e-03	1.01
HNSC	1.1e-02	1.00
KICH	6.8e-05	1.05
LGG	6.1e-07	1.01
LIHC	5.8e-04	1.01
LUAD	1.9e-02	1.00
MESO	1.4e-02	1.01
PAAD	1.4e-03	1.01
THCA	2.0e-02	1.03
UVM	6.0e-03	1.01

### DNA methylation of CBX3 across cancers

**Fig. 4:**
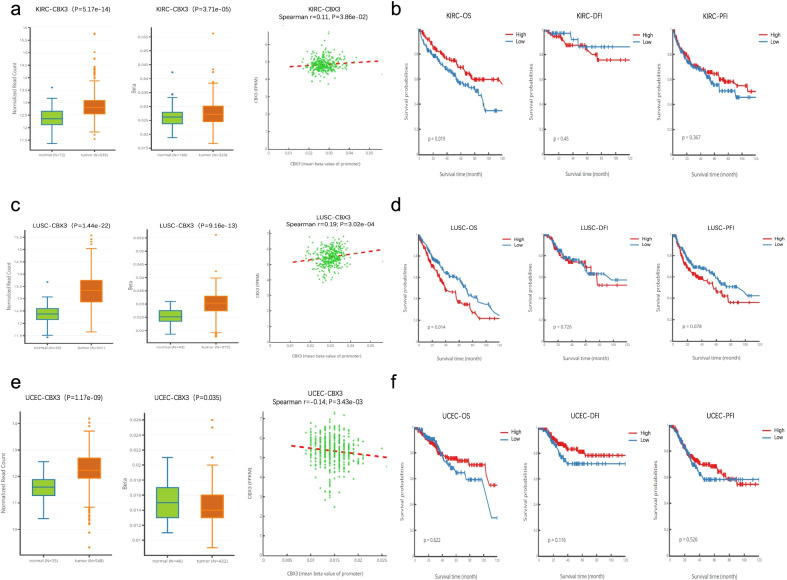
DNA methylation analysis of CBX3 gene in unequal tumors. The boxplot displayed the expression, DNA methylation for CBX3, and their spearman correlation in KIRC (**a**), LUSC (**c**), UCEC (**e**). The relationship between gene methylation of CBX3 and prognosis, including OS, DFI and PFI, in KIRC (**b**), LUSC (**d**), UCEC (**f**) were analyzed

The DNMIVD tool was used to investigate the potential association between DNA methylation and prognosis of pan-cancers based on TCGA and GEO databases. As shown in Table 5, 21 methylation sites of CBX3 and related information were summarized. Due to the limited number of tumors analyzed with the tools we used; only specific tumors were analyzed. The correlation between promoter methylation in KIRC, LUSC as well as UCEC and CBX3 gene expression were shown in Fig. 4a, c, e, respectively. Except in UCEC, we observed a significant positive correlation of CBX3 DNA methylation and gene expression in KIRC and LUSC. The results of this analysis suggest that there may be other mechanisms to increase the expression level of CBX3 and further experiments are needed to explore them. Furthermore, the Fig. 4b, d, f displayed the relationship between gene methylation and prognosis in KIRC, LUSC, and UCEC, respectively. In contrast to that in LUSC, the high methylation expression of CBX3 in KIRC showed a worse prognosis than the low expression.

**Table 5:** DNA methylation of CBX3

Gene symbol	CpG	Group	Relation to Island
CBX3	cg00485380	TSS1500	Island
CBX3	cg01027929	1stExon;5'UTR	Island
CBX3	cg02628353	5'UTR	Island
CBX3	cg03547487	TSS1500	Island
CBX3	cg03711622	TSS200;TSS1500	Island
CBX3	cg05827943	TSS200;1stExon;5'UTR	Island
CBX3	cg05857643	Body	S_Shore
CBX3	cg10141938	TSS1500	Island
CBX3	cg11010242	TSS1500	Island
CBX3	cg11844827	TSS1500	Island
CBX3	cg12647142	TSS200;TSS1500	Island
CBX3	cg13351698	TSS1500	Island
CBX3	cg14565914	3'UTR	OpenSea
CBX3	cg17545334	TSS200;TSS1500	Island
CBX3	cg17671317	TSS200;TSS1500	Island
CBX3	cg19658926	TSS1500	Island
CBX3	cg20706192	TSS200;TSS1500	Island
CBX3	cg21428393	Body	S_Shelf
CBX3	cg21815882	TSS200;TSS1500	Island
CBX3	cg24274982	TSS1500	Island
CBX3	cg26451575	TSS1500	Island

### Genetic alteration differences of CBX3 in cancers

**Fig. 5:**
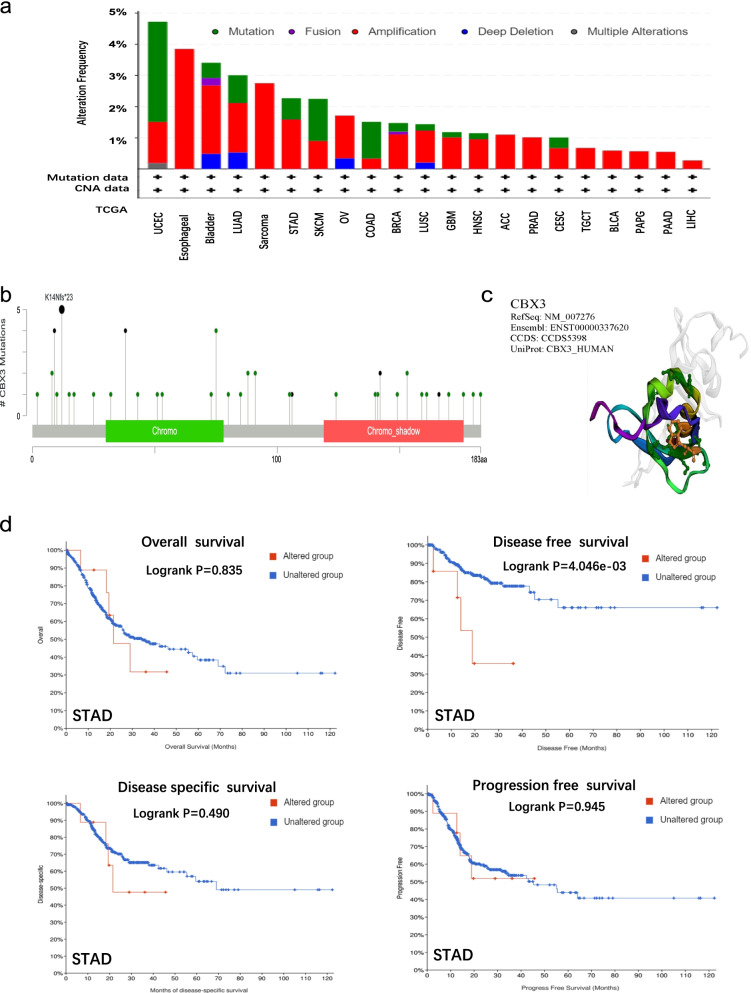
Mutation feature of CBX3 in different tumors of TCGA. The cBioPortal tool was used to analyze the mutation features of CBX3 for the TCGA tumors. The alteration frequency with mutation type (**a**) and mutation site (**b**) are displayed. We display the mutation site with the highest alteration frequency (K14Nfs*23) in the 3D structure of CBX3 (**c**). We also analyzed the potential correlation between mutation status and overall, disease-specific, disease-free, and progression-free survival of STAD (**d**) with the cBioPortal tool

The genetic variation of CBX3 in various cancer samples were observed via the cBioportal tool. As shown in Fig. 5a, the patients with UCEC occupied the highest alteration frequency of CBX3, nearly 5%. Furthermore, the primary type of alteration performed “mutation” (~ 3% frequency). The “amplification” type of CNA was the primary type in the esophageal cancer cases, which show an alteration frequency of ~ 4%. In addition, all sarcoma cancer cases with genetic alteration (~ 3% frequency) presented “amplification” alteration. As displayed in Fig. 5b, the types, sites, and case number of genetic alterations were presented clearly. We found that missense mutation of CBX3 was the primary type of alteration, and K14Nfs*23 alteration in the Chromo, which was detected in 2 cases of UCEC, 2 of STAD, and 1 of COAD. We presented the 3D structure of the CBX3 protein (Fig. 5c). Additionally, we explored the potential association between genetic alteration of CBX3 and the clinical survival prognosis of cases with different types of cancer. The data of Fig. 5d indicated that STAD cases with altered CBX3 showed poorer prognosis in disease-free survival (*P* = 4.046e-03) compared with ones without CBX3 alteration. However, no significant differences were observed in overall (*P* = 0.835), disease-specific (*P* = 0.490), and progression-free (*P* = 0.945) survival.

### The difference of protein phosphorylation levels in CBX3

**Fig. 6:**
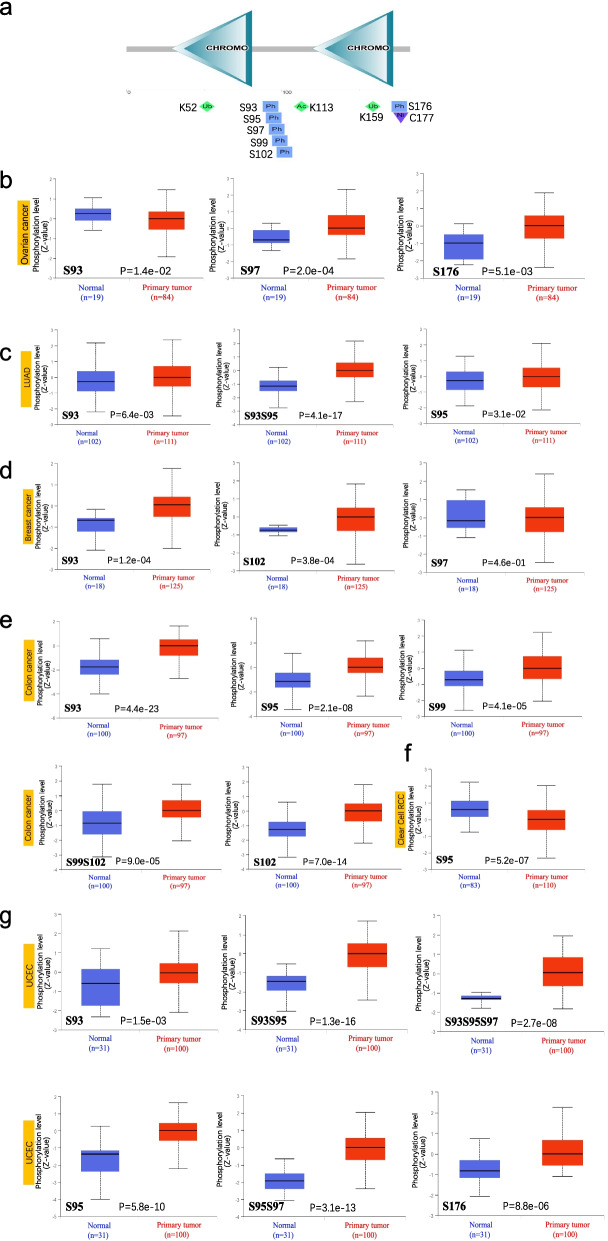
Phosphorylation analysis of CBX3 protein in multiple tumors. Based on the UniProt and SMART tools, we analyzed the protein domain of CBX3 phosphoprotein (S93, S95, S97, S99, S102, and S176 sites). The phosphoprotein sites with positive results are also displayed in the schematic diagram of CBX3 protein between normal tissue and primary tissue of selected tumors via the UALCAN (**a**). We also supply the box plots for different cancers, including ovarian cancer(**b**), LUAD (**c**), breast cancer (**d**), colon cancer (**e**), clear cell RCC (**f**), and UCEC (**g**)

The CBX3 phosphorylation sites from SMART web were summarized in the pattern diagram of Fig. 6a. The differences in protein phosphorylation levels between normal tissues and primary tumors were compared from the CPTAC dataset via UALCAN tool. Among them, we analyzed six types of tumors, including breast cancer, LUAD, clear cell RCC, ovarian cancer, colon cancer, and UCEC. The S93 locus of CBX3 exhibits a significant difference in phosphorylation level in five primary tumor tissues except for clear cell RCC, compared with normal tissues (Fig. 6b–g, all *P* < 0.05). In contrast with ovarian cancer, the other four cancers expressed higher phosphorylation levels than corresponding controls (Fig. 6b). The second change was an increased phosphorylation level of the S95 locus for LUAD (*P* = 3.1e-02), colon cancer (*P* = 2.1e-08), and UCEC (*P* = 5.8e-10). While, clear cell RCC (*P* = 5.2e-07) displayed decreased level. We also analyzed and summarized the expression difference in other phosphorylation sites. As shown in Fig. 6, most of the primary tumors displayed a higher phosphorylation level than the corresponding normal tissues.

### Correlation between CBX3 expression and TME across cancers

The TME contains various cells, yet infiltrating immune cells account for a large proportion [23, 24]. As prominent components of TME, these cells were closely related to the initiation, progression, or metastasis of cancer [25]. It was reported that the TILs and CAFs participated in modulating the function of various tumor-infiltrating immune cells [26]. Therefore, it is necessary to evaluate the correlation between CBX3 and the characters of immune infiltration in pan-cancer. In this part, we investigated the potential relationship between the infiltration level of different immune cells and CBX3 gene expression or clinical outcome in diverse cancer types of TCGA.

**Fig. 7:**
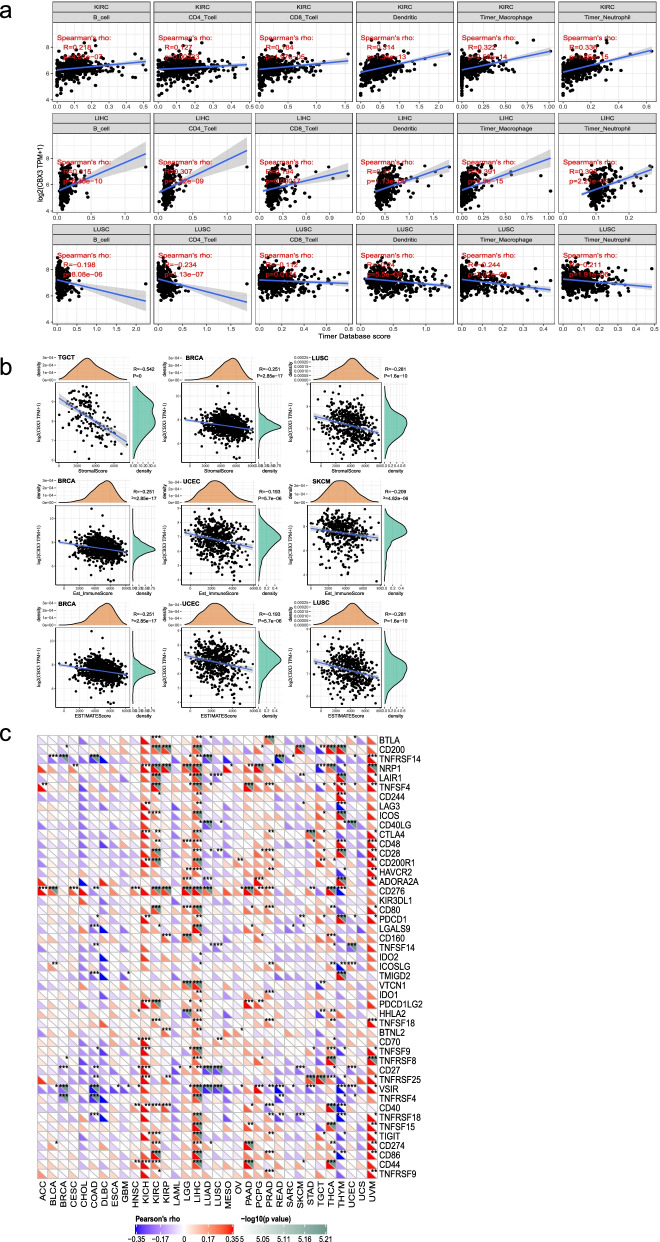
Correlation analysis between CBX3 and TME with the help of Sangerbox. **a** The correlation between CBX3 expression and TILs was shown in the top 3 cancers. **b** The scatter plots presented the relationship between CBX3 expression and the StromalScore, Est_ImmuneScore, ESTIMATEScore in the top 3 cancers. **c** The correlation between gene expression and immune checkpoint marker was displayed

We obtained the scores of 6 infiltrating immune cells from the TCGA database and analyzed the correlation with CBX3. As shown in Fig. 7a, results revealed that CBX3 expression was appreciably positively correlated with the infiltration levels of 6 immune cells in KIRC and LIHC, while negatively in LUSC. For the StromalScore, the top 3 cancers with significant differences showed negative correlations with CBX3 expression in TGCT, BRCA, and LUSC. The increased gene expression was correlated with decreased Est_ImmuneScore in BRCA, UCEC and SKCM. In addition, CBX3 expression was negatively correlated with the ESTIMATEScore in BRCA, UCEC and LUSC (Fig. 7b). Subsequently, we analyzed the correlation between CBX3 expression and that of 40 common immune checkpoint genes. The heat plot showed CBX3 expression was correlated with more than 20 immune checkpoint markers in UVM and KIRC. While, in LIHC, it had even close to 40 checkpoint markers (Fig. 7c). In short, these results strongly suggest that CBX3 plays a vital role in tumor immunity. Tumor mutational burden (TMB) is usually measured by the number of somatic mutations (non-synonymous mutations) in the average 1 Mb base of the coding region (exon region) of the genome of tumor cells. TMB is used to reflect the number of mutations in tumor cells and is a quantifiable biomarker. TMB is a recognized predictor of immunotherapy. Patients with higher TMB are more likely to benefit from immunotherapy. We also studied the relationship between the expression of CBX3 and TMB, and the results suggested that CBX3 were significantly positively correlated with TMB in BLCA, STAD and PRAD (Additional file 2: Fig. S5). It was suggested that CBX3 may be the target of immunotherapy in these tumors.

**Fig. 8:**
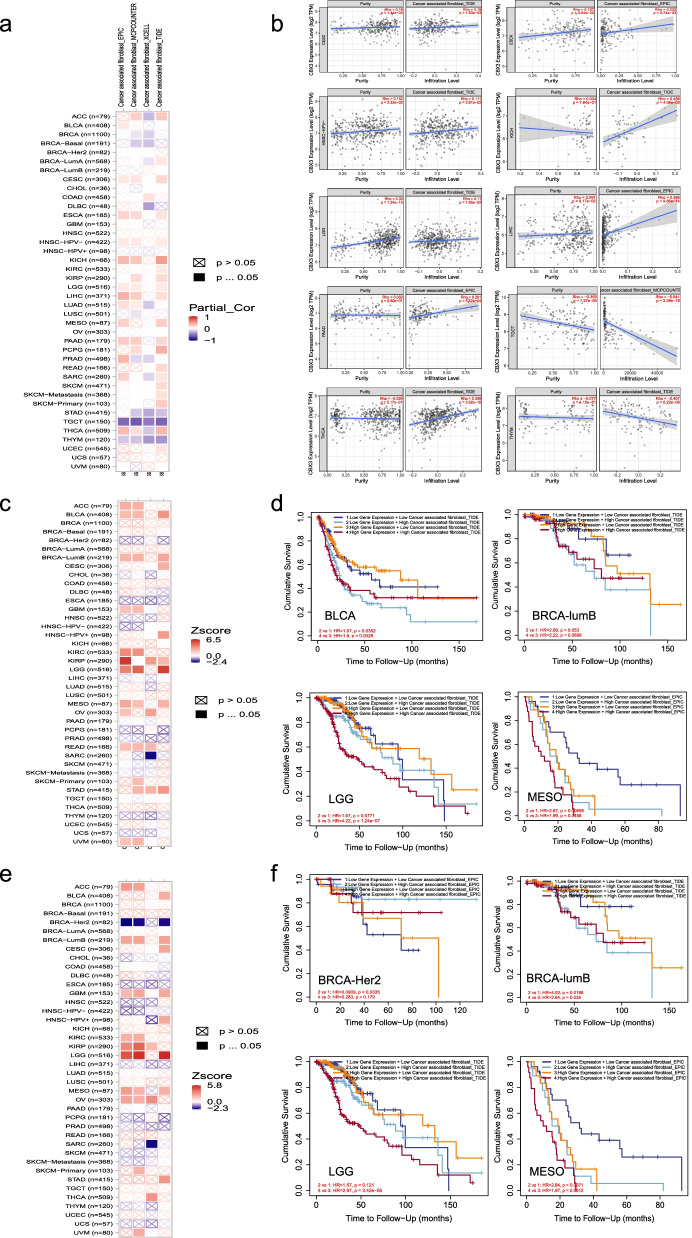
Correlation analysis between CBX3 and CAFs in TIMER2.0. The heat map (**a**) and the scatter diagram (**b**) of the correlation between CBX3 expression and infiltration of CAFs were illustrated, respectively. The heat map (**c**) and the survival curve (**d**) indicated the correlation between the clinical outcome of age and infiltration of CAFs. The figures **e** and **f** showed the relation on clinical stage and infiltration of CAFs

Moreover, we observed a positive statistical correlation of CBX3 expression and the estimated infiltration value of CAFs for the CESC, ESCA, HNSC-HPV-, KICH, LGG, LIHC, PAAD, and THCA. Still, we noted a negative correlation for TGCT and THYM (Fig. 8a) based on the TIDE, MCP-COUNTER and EPIC algorithms. The scatterplot data of the above tumors produced using one algorithm are presented in Fig. 8b. For instance, the CBX3 expression level in KICH is positively correlated with the infiltration level of CAFs (cor = 0.486, *P* = 4.06e-05) based on the TIDE algorithm. Similarly, as shown in Fig. 8c, d, we revealed the potential relationship between the cancer-associated fibroblast level and the clinical outcomes of age or stage in various cancer types in heat maps, respectively. In addition, the cumulative survival graphs on the cancer with statistical difference were displayed in Fig. 8e, f. For example, as the age of LGG patients with high infiltration levels increases, the survival outcome worsens. To sum up, the results indicated that CBX3 expression was closely correlated with the TME in cancers.

### Correlation analysis of CBX3-binding hub genes

**Fig. 9:**
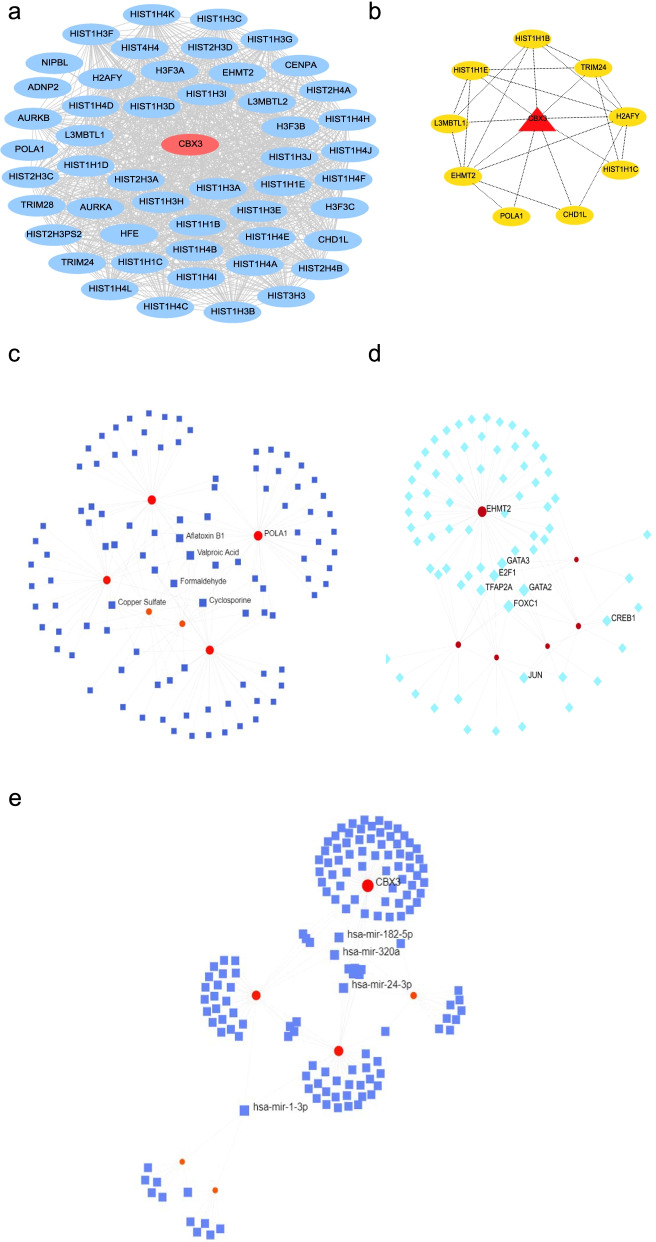
Correlation analysis of CBX3-binding hub genes. **a** We first obtained 50 available experimentally determined CBX3-binding genes using the STRING tool. **b** 10 CBX3-binding hub genes were analyzed with Cytohubba plug-in component in Cytoscape 3.8.0 software. We did interaction analysis of hub genes with chemical (**c**), transcription factor (**d**), and miRNA (**e**) based on the NetworkAnalyst approach

To further investigate the molecular mechanism of the CBX3 gene in the occurrence and development of tumors, we attempted to screen out and analyze the CBX3-binding hub genes. Based on the STRING tool, we obtained 50 CBX3-binding genes, supported by experimental evidence (Fig. 9a). Afterward,10 hub genes (CBX3, TRIM24, HIST1H1E, HIST1H1C, H2AFY, CHD1L, POLA1, EHMT2, L3MBTL1, HIST1H1B) were further screened with the help of the Cytohubba module (Fig. 9b).

In the protein-chemical interactions network of Fig. 9c, hubs were associated with the chemical substances, involving Aflatoxin B1, Valproic Acid, Formaldehyde, Copper Sulfate, Cyclosporine. As a predisposing factor, these toxic chemicals, may induce cancer by affecting CBX3 or its binding genes.

We presented hubs-TFs interactions (Fig. 9d) and hubs-miRNAs interactions (Fig. 9e) in network diagrams, respectively. We further detected central regulatory biomolecules (TFs and miRNAs) according to the set topological parameters. Seven TFs (GATA3, E2F1, TFAP2A, GATA2, FOXC1, JUN, CREB1) and four miRNAs (Hsa-mir-182-5p, Hsa-mir-320a, Hsa-mir-24-3p, Hsa-mir-1-3p) were detected from the hubs-TFs and hubs-miRNAs interaction networks, respectively. The TFs and miRNAs participated in the tumorigenesis by regulating CBX3-binding hub genes at the transcription level and post-transcriptional level.

### Enrichment analysis of CBX3-related partners

**Fig. 10:**
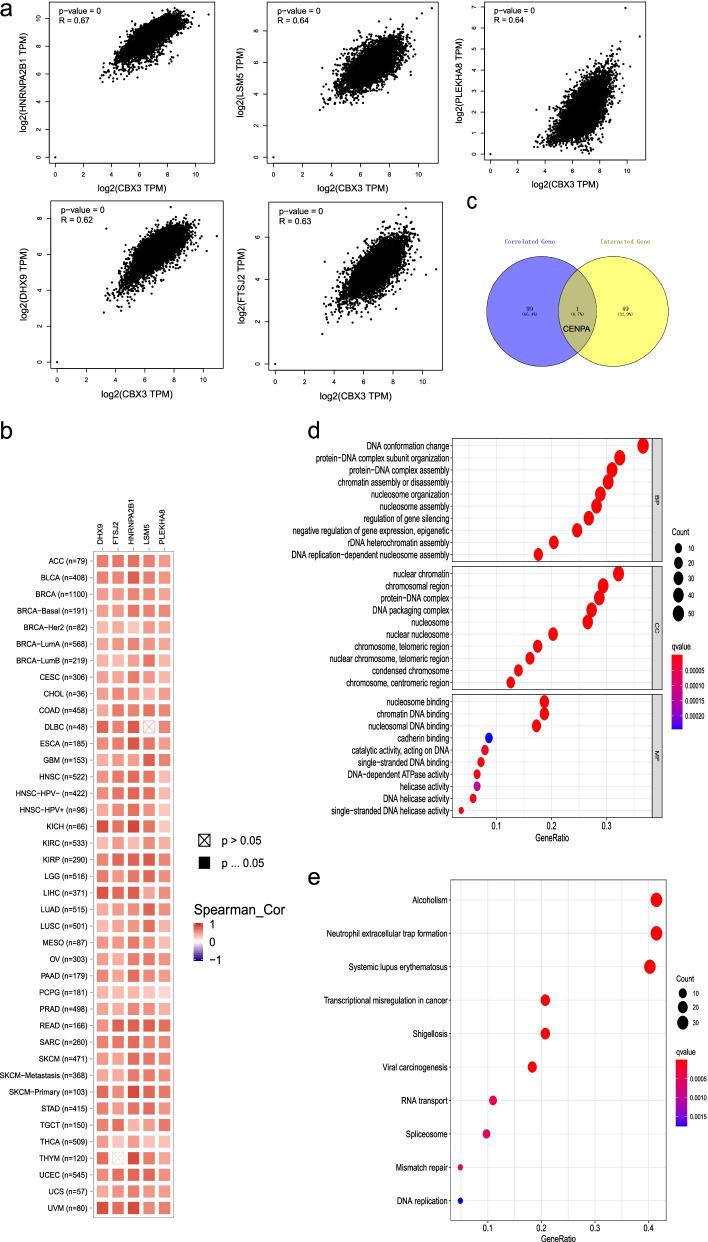
CBX3-related gene enrichment analysis. **a** Based on the GEPIA2 approach, we obtained the top 100 CBX3-correlated genes in TCGA projects and analyzed the expression correlation between CBX3 and selected targeting genes, including HNRNPA2B1, PLEKHA8, LSM5, FTSJ2, and DHX9. **b** The heatmap of selected target genes in detailed cancer types were displayed. **c** An intersection analysis of CBX3-binding and correlated genes was conducted in a Venn diagram. Based on the CBX3-binding and interacted genes, the bubble chart for the molecular function data in GO analysis (**d**) and KEGG pathway analysis (**e**) were performed with R 4.0.2 software

Similarly, the CBX3 expression-correlated genes were also screened out for pathway enrichment analyses to investigate the molecular mechanism from another view. We screened the top 100 genes that correlated with CBX3 expression with the GEPIA2 tool. The scatter plots (Fig. 10a) displayed the positive correlation between CBX3 expression level and that of HNRNPA2B1(R = 0.67), PLEKHA8(R = 0.64), LSM5(R = 0.64), FTSJ2(R = 0.63) and DHX9(R = 0.62) genes (all *P* < 0.001). Moreover, the corresponding heatmap further confirmed the positive association between CBX3 and the five genes in most tumors (Fig. 10b). We did an intersection analysis of the correlated gene group and interacted gene group, then obtained one common member, centromere protein A CENPA (Fig. 10c).

The GO enrichment analysis data were presented in three parts, including BP (Biological Process), CC (Cellular Component), and MF (Molecular Function). The results indicated that most genes are linked to the cellular biology of DNA. For instance, DNA conformation change and protein-DNA subunit organization played a major role in the BP part. In the CC part, nuclear chromatin and chromosomal region were the main enrichment positions. The changes in molecular function of nucleosome binding and chromatin DNA binding were linked to the pathways (Fig. 10d). The bubble plot of KEGG data suggested that the two pathways of “alcoholism” and “neutrophil extracellular trap formation” might be involved in the tumor pathogenesis of CBX3 to a large extent (Fig. 10e).

## Discussion

The pan-cancer analysis of genes are of importance for the prevention of cancer, the development of biomarkers, and the search for therapeutic target [27]. It has been reported that CBX3 participates in cellular biological processes, such as chromosome segregation, transcriptional regulation, DNA repair, and RNA splicing [28]. Increased literature reported high expression of CBX3 can induce the development of multiple cancers, and was associated with poor prognosis [29–31]. The CBX3 gene played a tumor-promoting role in PAAD and induced the increased proliferation, migration, and invasion of the PAAD cells. Similarly, the silence of CBX3 can inhibit glioma cell proliferation by blocking the cell cycle in the G2/M phase [32]. The CBX3 may function as a tumor promoter. Whether CBX3 can play a crucial role in the pathogenesis of multiple tumors has not been identified. Thus, the molecular features in diverse expression patterns, genetic alteration, protein phosphorylation, prognostic values, and immune infiltration of CBX3 were examined by comprehensive bioinformatics.

In this study, we found that abnormal expression of CBX3 existed in human pan-cancer, including BRCA, COAD, PAAD, UCEC, and LUAD. Nevertheless, the survival prognosis analysis data from different forms of prognosis for the CBX3 gene suggested distinct conclusions for various tumors. In multiple cancers, survival analysis showed CBX3 overexpression was associated with poor prognosis, especially in CESC, LIHC, LUAD, and PAAD. The result was consistent with a recent article on CBXs and LUAD from Kaplan–Meier analysis [33]. The result of previous research indicated CBX3/HP1γ was the most frequently overexpressed and amplified one, and high mRNA levels were associated with poor prognosis in LUAD patients [34]. In addition, further regression model analysis showed the role of independent prediction of CBX3 for OS and PFI in LUAD. Similarly, CBX3 was found to be overexpressed in PAAD based on the data from GEO datasets. Furthermore, for the proliferation and invasiveness of PAAD cells in vitro, CBX3 played a role in acceleration. The high expression level was also associated with a poor prognosis of overall and disease-free survival [35]. The different data sources and survival information both demonstrated similar results. To sum up, our data strongly indicate CBX3 may be a potential prognostic biomarker and molecular therapeutic target in LUAD and PAAD.

For cancers in the digestive system, the relationship between gastric cancer, hepatocellular carcinoma, colorectal cancer and CBX3 were analyzed, besides PAAD. An article reported the overexpression of CBX3 was significantly correlated with a short OS prognosis in STAD [36]. However, another study has shown the OS prognosis was better in STAD patients with high CBX3 expression. In our results, we failed to observe a significant correlation. The diversity of sample numbers and grouping method in the prognostic analysis may be the reason. Thus, larger sample sizes are required to confirm the effect of CBX3 in the survival prognosis of STAD. CBX3 showed a mutation rate of more than 5% in STAD [37]. Our results also observed a mutation in the site of K14Nfs*23. Furthermore, the variation was negatively correlated with disease free survival in patients with STAD. Based on Kaplan–Meier and Cox regression analyses, other research [38] verified our survival analysis result about the correlation between CBX3 high expression and poor clinical prognosis of LIHC. However, the previous one just examined the overall survival. Our study further analyzed the remaining prognostic model, including PFI, DFI and DSS.

HP1 family proteins are extensively modified in a manner analogous to histones, including phosphorylation, acetylation, and methylation. The modification can modulate their sub-nuclear location and bioactivity [39]. HP1γ can specifically bind methylated histone H3, such as H3K36, through their conserved chromodomain [40]. Furthermore, data analysis indicated multiple phosphorylation sites existed on HP1γ. However, there is almost no research about the influence of protein modification on tumors. We first used the DNMIVD and CPTAC datasets to explore the CBX3 methylation and phosphorylation expression level across all TCGA cancers. The findings indicated the correlation between methylation and expression, prognosis, or phosphorylation level of CBX3 in some tumors. Further, additional evidence is required to evaluate CBX3 methylation and phosphorylation’s potential role in tumorigenesis.

It has been reported that immune cells in TME played a role in either tumor-promoting or tumor-suppressing. And they were associated with the proliferation and progression of cancer cells. Our results first suggested that CBX3 expression was significantly associated with the TILs and cancer—associated fibroblast in certain tumors. They implied that CBXs may potentially reflect the immune status besides the prognosis. At present, there are few studies and literature on CBX3 and immune infiltration. There is a research report suggesting CBX3 affects factors related to immunotherapy in STAD [41]. These results were in line with our data in TILs and TMB (Additional file 1: Fig. S3, Additional file 2: Fig. S5). The previous finding showed that CBX3 was positively associated with CD8 + T cells and macrophages infiltration in COAD [42]. Similarly, our observation (Additional file 1: Fig. S2) was consistent with it. In addition, we also observed a statistical correlation between CBX3 expression and the immune infiltration level of TILs in the tumors of KIRC, LIHC, and LUSC. Besides, other immune-associated indexes in multiple cancers were examined, including immunization score, matrix score, check point, neoantigen (Additional file 2: Fig. S4), and TMB. These findings might present detailed immunization evidence across all TCGA tumors to assist for new immunotherapies.

Furthermore, we integrated the information on CBX3-binding genes and CBX3-related partners for a series of analyses and obtained one common component, CENPA. The identified TFs and miRNAs participated in tumorigenesis by regulating CBX3-binding hub genes at the transcription and post-transcriptional levels. The related chemical substances, change in the cellular biology of DNA and other enriched pathways have a potential impact on the etiology and molecular mechanism. The CENPA is an epigenetic mark that determines centromere identity. Existing research reported overexpression of the gene is crucial to specific cancer growth [43]. The expression of CENPA was estimated across all tumors from TCGA and GTEx database (Additional file 2: Fig. S6). Interestingly, all showed a statistical difference compared to the corresponding normal group. It implied the analytical value for biomarkers or therapeutic target in pan-cancers.

However, even though we systematically investigated information on pan-cancer from multiple databases, there were inevitable limitations in this study. Most importantly, additional validation experiments are needed to carry out clinical samples or appropriate models in vitro or in vivo. Further research will contribute to clarifying the role of CBX3 at the molecular level. In addition, the finding showed that CBX3 expression was correlated with tumor immunity and clinical survival. However, deep studies focusing on CBX3 expression and tumor immune microenvironment should be researched to provide a therapeutic strategy based on the immune.

## Conclusion

Taken together, the first pan-cancer analyses of CBX3 indicated statistical correlations of CBX3 expression with survival prognosis, DNA methylation, protein phosphorylation, immune cell infiltration across multiple tumors. Because CBX3 showed overexpression in multiple cancers and correlation with poor prognosis, CBX3 might be considered a novel biomarker and therapeutic target. In addition, our analysis provided a potential mechanism that CBX3 might regulate the tumor immune microenvironment. The results contribute to explaining the role of CBX3 in tumorigenesis from the perspective of clinical tumor samples.

## Supplementary Information

**Additional file 1:** CBX3 expression with no difference in certain tumor tissues and the correlation between CBX3 with TILs in STAD/COAD.

**Additional file 2:** The relationship between CBX3 with immune-associated indexes (neoantigen, TMB) and expression difference of CENPA.

## Data Availability

The authors ensure that the data analyzed in the research is publicly available. The data can be found as follows: TIMER2.0 (http://timer.cistrome.org/), UALCAN (http://ualcan.path.uab.edu/), cBioPortal (https://www.cbioportal.org/), CCLE (https://portals.broadinstitute.org/ccle), GEPIA2 (http://gepia2.cancer-pku.cn/#analysis), Human Protein Atlas (https://www.proteinatlas.org/), DNMIVD (http://119.3.41.228/dnmivd/index/), ONCOMINE (https://www.ONCOMINE.org/).

## Abbreviations

ACC: Adrenocortical carcinoma
BLCA: Bladder urothelial carcinoma
BRCA: Breast invasive carcinoma
CAFs: Cancer-associated fibroblasts
CBX3: Chromobox protein homolog 3
CENPA: Centromere protein A
CESC: Cervical squamous cell carcinoma and endocervical adenocarcinoma
CHOL: Cholangiocarcinoma
COAD: Colon adenocarcinoma
DFI: Disease-free interval
DLBC: Lymphoid neoplasm diffuse large B-cell lymphoma
DSS: Disease-specific survival
ESCA: Esophageal carcinoma
GBM: Glioblastoma multiforme
HNSC: Head and neck squamous cell carcinoma
KICH: Kidney chromophobe
KIRC: Kidney renal clear cell carcinoma
KIRP: Kidney renal papillary cell carcinoma
LAML: Acute myeloid leukemia
LGG: Brain lower grade glioma
LIHC: Liver hepatocellular carcinoma
LUAD: Lung Adenocarcinoma
LUSC: Lung squamous cell carcinoma
OS: Overall survival
OV: Ovarian serous cystadenocarcinoma
PAAD: Pancreatic adenocarcinoma
PCPG: Pheochromocytoma and paraganglioma
PFI: Progression-free interval
PRAD: Prostate adenocarcinoma
SARC: Sarcoma
SKCM: Skin cutaneous melanoma
STAD: Stomach adenocarcinoma
TGCT: Testicular germ cell tumors
THCA: Thyroid carcinoma
THYM: Thymoma
TIL: Tumor-infiltrating lymphocyte
TMB: Tumor mutational burden
UCEC: Uterine corpus endometrial carcinoma
UCS: Uterine carcinosarcoma
HNSC-HPV: Head and neck squamous carcinoma- HPV
HR: Hazard ratio
