# Supplementary material for: Comprehensive pan-cancer analysis on CBX3 as a prognostic and immunological biomarker
Source: BMC Med Genomics. 2022 Feb 16;15:29. doi: 10.1186/s12920-022-01179-y (PMC8851738; doi:10.1186/s12920-022-01179-y)
Supplement: Supplementary file 1 — Additional file 1: CBX3 expression with no difference in certain tumor tissues and the correlation between CBX3 with TILs in STAD/COAD. [file 12920_2022_1179_MOESM1_ESM.pdf]

S1

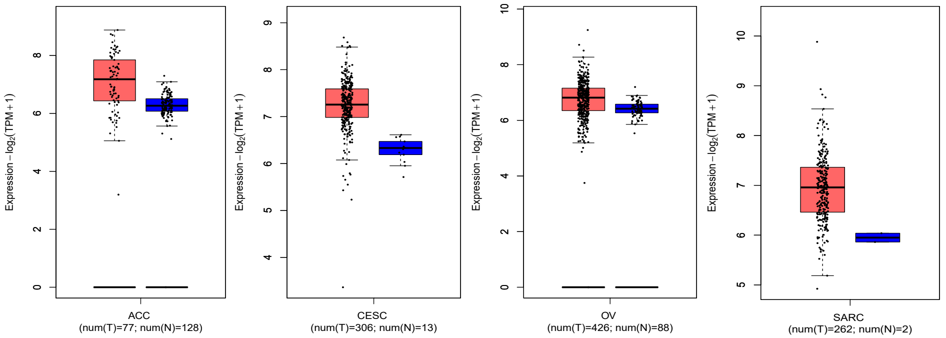

S1: The box plots of ACC, CESC, OV, and SARC in TCGA were supplied, the corresponding normal tissues from the GTEx database.

S2

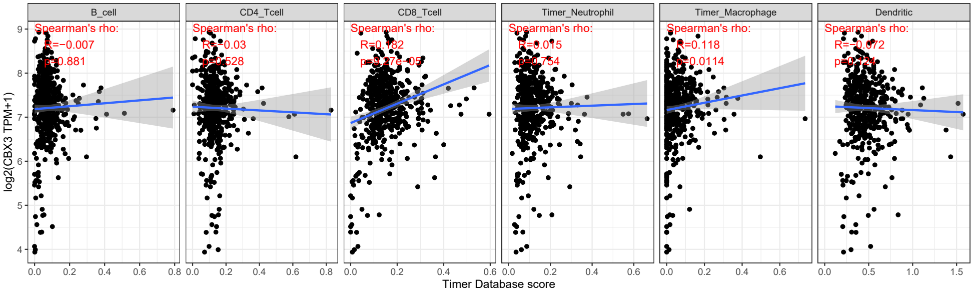

S2: The scatter plots displayed the correlation between CBX3 expression and TILs in COAD.

S3

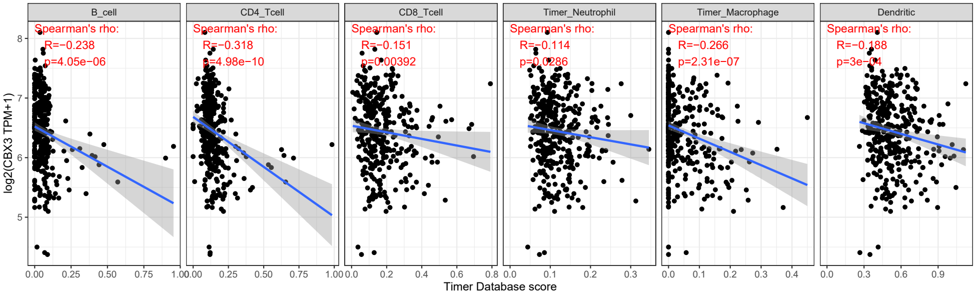

S3: The scatter plots displayed the correlation between CBX3 expression and TILs in STAD.
