# Supplementary material for: Comprehensive pan-cancer analysis on CBX3 as a prognostic and immunological biomarker
Source: BMC Med Genomics. 2022 Feb 16;15:29. doi: 10.1186/s12920-022-01179-y (PMC8851738; doi:10.1186/s12920-022-01179-y)
Supplement: Supplementary file 2 — Additional file 2: The relationship between CBX3 with immune-associated indexes (neoantigen, TMB) and expression difference of CENPA. [file 12920_2022_1179_MOESM2_ESM.pdf]

S4

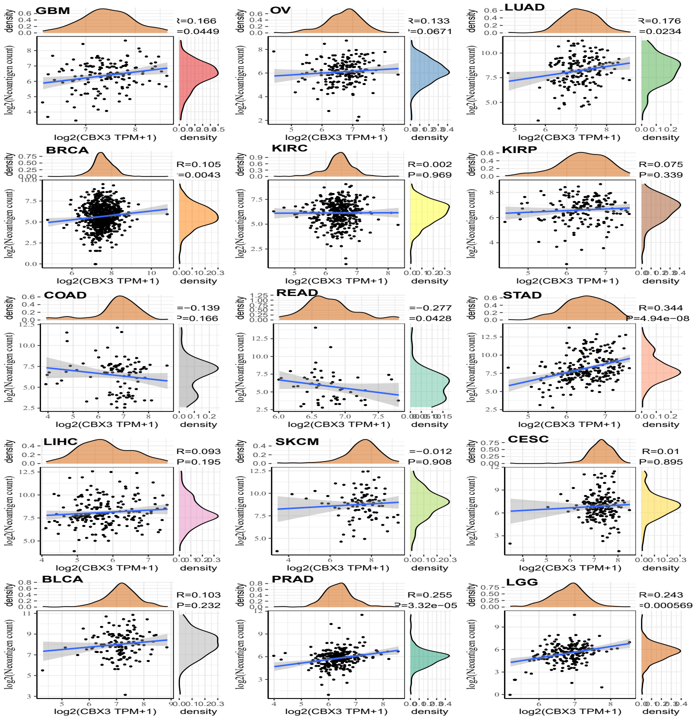

S4: The relationship between CBX3 expression and neoantigen in different tumors

S5

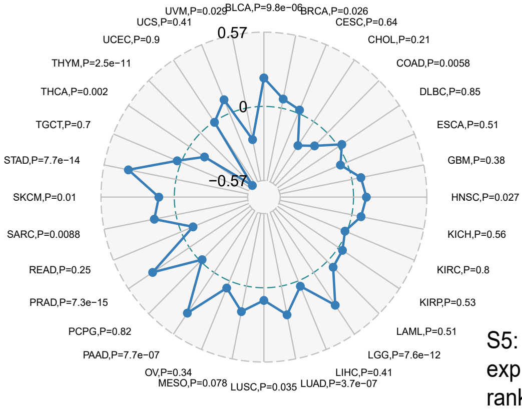

S5: The relationship between CBX3 expression and TMB with spearman rank correlation coefficient in radar chart

S6

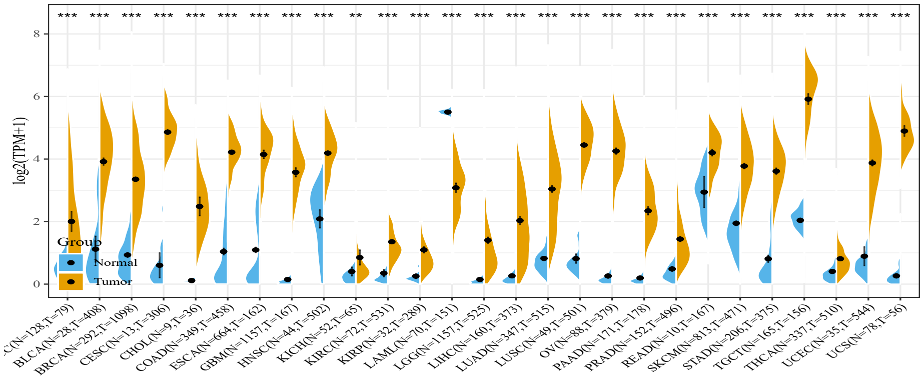

S6: The difference in CENPA expression between cancer and adjacent tissue in each tumor sample
